# Advanced biosensors for detection of pathogens related to livestock and poultry

**DOI:** 10.1186/s13567-017-0418-5

**Published:** 2017-02-21

**Authors:** Jasmina Vidic, Marisa Manzano, Chung-Ming Chang, Nicole Jaffrezic-Renault

**Affiliations:** 1 Virologie et Immunologie Moléculaires, UR892, INRA, Paris Saclay University, 78350 Jouy-en-Josas, France; 2 Dipartimento di Scienze AgroAlimentari, Ambientali e Animali, Università di Udine, 33100 Udine, Italy; 3 Department of Medical Biotechnology and Laboratory Science, College of Medicine, Chang Gung University, Tao-Yuan, 33302 Taiwan; 4 Institute of Analytical Sciences, UMR CNRS 5280, University of Lyon, 69100 Villeurbanne, France

## Abstract

Infectious animal diseases caused by pathogenic microorganisms such as bacteria and viruses threaten the health and well-being of wildlife, livestock, and human populations, limit productivity and increase significantly economic losses to each sector. The pathogen detection is an important step for the diagnostics, successful treatment of animal infection diseases and control management in farms and field conditions. Current techniques employed to diagnose pathogens in livestock and poultry include classical plate-based methods and conventional biochemical methods as enzyme-linked immunosorbent assays (ELISA). These methods are time-consuming and frequently incapable to distinguish between low and highly pathogenic strains. Molecular techniques such as polymerase chain reaction (PCR) and real time PCR (RT-PCR) have also been proposed to be used to diagnose and identify relevant infectious disease in animals. However these DNA-based methodologies need isolated genetic materials and sophisticated instruments, being not suitable for in field analysis. Consequently, there is strong interest for developing new swift point-of-care biosensing systems for early detection of animal diseases with high sensitivity and specificity. In this review, we provide an overview of the innovative biosensing systems that can be applied for livestock pathogen detection. Different sensing strategies based on DNA receptors, glycan, aptamers and antibodies are presented. Besides devices still at development level some are validated according to standards of the World Organization for Animal Health and are commercially available. Especially, paper-based platforms proposed as an affordable, rapid and easy to perform sensing systems for implementation in field condition are included in this review.

## Introduction

Infectious diseases are the leading causes of death of humans and animals worldwide. Wildlife and domestic animals pathogen infections threat animal production and food supply, seriously impact animal welfare and have potential environmental and global biodiversity consequences. There is a clear economic cost of animal infectious disease as they impact large-scale developmental projects. In addition, viral infections of animal population carry global public health risks of sporadic human zoonotic infections or emergence of a pandemic viral strain. Animals are thought to be the source of more than 70% of all emerging infections [1].

One of essential elements for implementation of an efficient response to infectious disease threats is a rapid, selective and sensitive assay for pathogen diagnostics. Current research attempts to adopt a multidisciplinary approach for both identification of underlying pathogenic agents and control infectious diseases spread. Certainly, early detection of pathogen is crucial for managing infections and establishing improved decision-making tools.

Conventional methods for viral detection include virus or microorganism propagation and isolation from culture. These methods are effective and sensitive but tend to be costly, labor intensive and time consuming (typically results are available in 2–10 days). Alternative molecular methods based on polymerase chain reaction (PCR), real time PCR (RT-PCR) are more specific, sensitive and take less time, but they need isolated genetic materials, manipulation with special care and necessitate sophisticated equipment, and, thus, they are hardly to be applied for on-site monitoring. Consequently, development of a valid diagnostic assay for swift pathogen detection and identification, with high sensitivity and selectivity is a challenge for researchers all over the world.

Biosensors, as analytical devices, are attractive solutions for fast and efficient infectious disease diagnostics due to their simplicity, possible miniaturization and potential ability for real-time analysis [2–6]. Over the past 30 years, a number of biotechnological innovations have provided biosensors for bacterial and viral detection and monitoring. Some emerging systems have resulted in promising prototypes that achieved rapid pathogen detection without demanding high level of sample manipulation which is highly inconvenient for infected samples.

In this review we will focus on biosensors that can be applied for domestic animal pathogen diagnostics. Different biomarkers of animal infectious diseases (as proteins, DNA, RNA) and commonly used in biosensing technologies, especially for virus detection are considered in details. Examples are given for pathogens responsible for major economic losses in cattle, pig, sheep and poultry farming.

## Principal of biosensing technology

**Figure 1:**
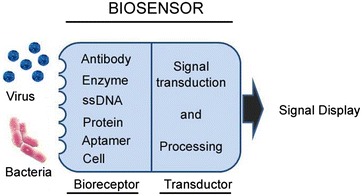
**Principle of biosensors.** A schematic diagram of pathogen detection by a biosensor.

Biosensor recognizes a target biomarker, characteristic for particular pathogen, via an immobilized sensing element called bioreceptor (monoclonal antibody, RNA, DNA, glycan, lectin, enzyme, tissue, whole cell). The bioreceptor is a crucial component as its biochemical properties assure high sensitivity and selectivity of the biomarker detection and permit to avoid interferences from other microorganisms or molecules present in the tested sample. The specific biochemical interaction between the biomarker and the bioreceptor is converted into a measurable signal by the transductor (Figure 1). Signal recording and display should, then, allow qualitative and quantitative pathogen identification.

There are two principal challenges to develop a biosensor for pathogen detection: (i) elaboration of a bioassay for biomarker detection, and (ii) improving the robustness of the bioassay to adapt it for applications in field and/or on complex biological samples. Indeed, many bioassays that work well on the bench with purified biomarker molecules fail to detect them in complex media like blood or serum. In addition, diagnostics of infection disease require high sensitivity since pathogens might spread rapidly before that any clinical sign appears in animals.

## Detection of *Escherichia coli*

*Escherichia coli* (*E. coli*) is a Gram-negative rod-shaped bacterium, diversified into harmless strains, normally found in the lower intestine microbiota of humans and animals, and virulent strains that cause infections, including gastroenteritis, urinary tract infection, meningitis, peritonitis, and septicemia. In poultry, *E. coli* causes colibacillosis characterized by the migration of the virulent strains, as O78:K80, O1:K1, and O2:K1, from intestine to other organs as respiratory or urinary tracts. These infections decreases egg production, reduce chicken grow, increase mortality and cause important economic losses. For instance, in 2011, an outbreak of a highly virulent *E. coli* in Europe resulted in subsequent food withdrawals from the market and export bans leading to about $417 million negative economic impact for EU farmers [7]. Colibacillosis is also seen in a variety of farm animals like cattle, pigs, goats [8] and has a significant economic importance concerning the loss of livestock. In cattle, pathogenic variants of *E. coli* are responsible for diseases, as septicemia and diarrhea in newborn calves or acute mastitis in dairy cows. The use of antibiotic in colibacillosis treatment and prevention is become an even greater problem than the infection itself. The extensive antibiotic use in animal production has incidence on the spread of multidrug-resistant bacteria and on antibiotic-resistant infections in humans. In most developed countries, serious consideration is being undertaken to regulate and severely restrict the use of antibiotics in animal production. The rapid and accurate diagnostic of virulent *E. coli* strains is vital for assessing the antibiotic resistance information, for administration of appropriate treatment and thus for avoiding useless antibiotics utilization.

The main issue in *E. coli* diagnostic is to distinguish between the closely related strains in order to distinguish between pathogenic and non-pathogenic species. Commonly used methods for *E. coli* detection and identification include culture, fermentation, enzyme linked immunosorbent and PCR assay. These methods show disadvantages in terms of long identification time (typically few days), high labor and reagent cost [9, 10]. Novel biosensors based on specific biochemical recognition strategies have been reported for rapid and specific *E. coli* detection as those based on PCR [11], quartz crystal microbalance system [12], surface plasmon resonance [13], chemiluminescence [14] and electrochemistry [15–17]. The biosensors for *E. coli* diagnostics are elaborated to assure two major steps: the capture of target bacteria from the biological or environmental samples and subsequent identification of captured bacterial sub-type. A huge variety of high affinity antibodies against *E. coli* that bind to surface/flagella proteins is available, as well as appropriate labels that can be employed for the amplification of detectable signal (as enzymes, biofunctionalized nanoparticles or fluorophores). These allow development of various sandwich-type immunosensors and immunoassays for rapid detection of *E. coli* in infected samples.

Immunosensor developed by Jaffrezic-Renault et al. [18] is based on the following strategy: addressable magnetic nanoparticles coupled with anti-LPS antibodies were used for the generic capture of Gram-negative bacteria onto the graphite ink electrode. The use of immunomagnetic beads allow detection of a biomarker contained in complex sample matrices. Conductometric measurements allowed real-time, sensitive detection of *E. coli* or *Serratia marcescens* cultures from 1 to 10^3^ CFU/mL. The conductometric immunosensor permitted also the direct detection of 10 to 10^3^ CFU/mL of *Pseudomonas aeruginosa* and *Acinetobacter baumannii* strains that were undetectable using standard immunoblot methods. Gram-positive bacteria such as *Staphylococcus epidermidis* were not detected indicating the specificity of detection [19].

Eltzov and Marks [20, 21] have proposed a point-of-care detection system based on stacked paper membranes that quantify *E. coli* within less than 5 min. When liquid samples containing bacteria are added onto the bottom membrane layer, the liquid starts to migrate from the lower to the upper layers. As each layer becomes wet, *E. coli* cells from contaminated samples push through to the next membrane layers. During migration, the bacterial cells bind with the specific antibody, itself conjugated with horseradish peroxidase (HRP) enzyme to allow signal measurement. In target-free samples, the HRP-labeled anti-*E. coli* antibodies from migrating are stopped by previously immobilized target bacteria on the capture layer. The upper-most layer contains the HRP enzymatic substrate producing a measurable signal only with samples containing target *E. coli*. This portable and rapid immunoassay was shown to have around 1000-folds higher sensitivity than ELISA since only 100 cells/mL were successfully quantified.

## Detection of avian influenza viruses

Aquatic birds constitute the main reservoir for avian influenza viruses (AIVs) [22]. These viruses represent a global threat to animal health and international poultry industry. Particularly high concern represents pandemic emergences which may cause enormous economic losses. AIVs are divided into low and highly pathogenic strains regarding their pathogenicity for chicken. The highly pathogenic AIV (HPAIV) spreads rapidly in domestic poultry and result in high mortality rate. The low pathogenic AIV (LPAIV) may cause mild respiratory or gastrointestinal symptoms, but usually without any signs of illness. However, LPAIV strains may acquire high pathogenicity during multiple infections in a chicken population [23].

Based on their antigenic specificity, Influenza A viruses infecting birds, are divided into 16 hemagglutinins (HA, H1-16) and 9 neuraminidases (NA, N1-9) subtypes. To date, naturally occurred HPAIV that produce high mortality in chickens, turkeys and other birds of economic importance have been restricted only to H5, H7 and H9 subtypes. Of these viruses, some H5N1, H7N2 and H7N7 viruses have shown to cause up to 100% mortality with 48 h in infected chickens. Many outbreaks of HPAIV occurred in domestic poultry production systems as it was the case with the highly pathogenic avian influenza H5N8 virus since 2014 [24]. H5N1 viruses have affected the poultry industry in numerous countries for the past 15 years and have resulted in the deaths of millions of birds. Consequently, a global influenza virological surveillance in poultry populations and migratory birds is recommended by both by World Organization for Animal Health (OIE) and World Health Organization (WHO) [25, 26]. Availability of a swift and sensitive AIV diagnosis tool that allows virus strain identification will be highly useful for disease surveillance as well as for optimizing biosecurity measures on farms.

Avian influenza virus belong to the Influenza A virus family *Orthomyxoviridae*. The genome of AIV is constituted by eight segments of negative-stranded RNA. Ten main proteins are encoded by viral genome: the polymerase basic protein 1 (PB1) and 2 (PB2), hemagglutinin (HA), nucleoprotein (NP), neuraminidase (NA), the polymerase acidic protein (PA), matrix proteins 1 and 2, and non-structural protein 1 and 2 [27]. Some other influenza A virus proteins were found as PB1-F2, PB1-N40, PA-X, NEP, M42, PA-N155, PA-N182 [28–31]. Potentially all these viral proteins, as well as their corresponding coding RNA sequences, are AIV biomarkers. Nevertheless, frequent mutations in the AIV genome lead to changes in antigenic properties which restrained the choice of biomarkers. HA, M2 and NA are the dominant targets for the host antibody response, anti-viral drug development and represent also the targets in diagnostics assays.

A broad of traditional serological diagnostic methods like haemagglutination (HA) test, Hemagglutination-inhibition (HI) test, neutralization (NT) and ELISA test are available as well as those based on virus propagation and isolation from cell culture or embryonated chicken eggs. These methods are effective and sensitive but they require relatively important amount of virus particles and special sample collection and handling tend to be costly, labor intensive and time-consuming. Moreover, HPAIV, as H5N1, are quite virulent for eggs, killing them quickly. This makes standard egg culture amplification procedure quite difficult. Alternative molecular methods based on PCR and RT-PCR are more sensitive but need extracted genetic material and require the use of equipment which is available only in diagnostic or scientific laboratories. In consequence, on-site detection of avian influenza viruses is rare until now for both early diagnostics and monitoring [32–34]. Similarly, a variety of different serological tests that detect the response of the infected host are established but they are not adapted to strains which have a pandemic potential. Moreover they are not robust and are difficult to be employed in point-of-care condition [35]. There are several bedside tests which allow a relatively quick (up to 30 min) detection of viral antigens [36]. Unfortunately, these tests provide low sensitivity and often produce false negative results, especially during later stages of the disease development. Low sensitivity is the main reason why these tests are rarely used in routine diagnostics of influenza virus.

**Figure 2:**
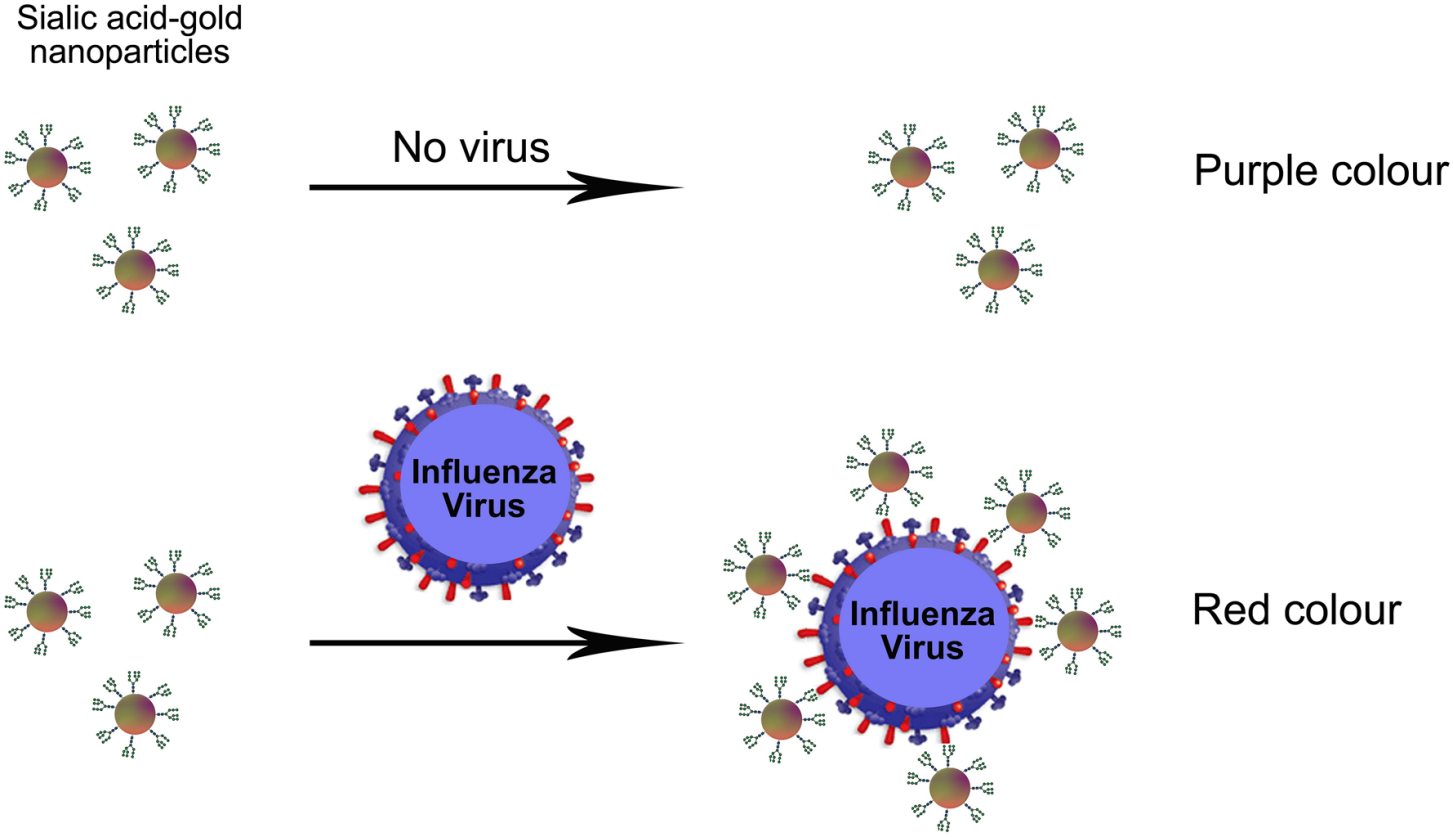
**Colorimetric sensor for detection of influenza A virus.** Sialic-mediated colorimetric detection of Influenza virus. Gold nanoparticles are stabilized with sialic to specifically bind HA protein on Influenza virus surface. Sialic-acid gold nanoparticles alone show the absorbance at 510 nm, while virus-bound nanoparticles absorbed at 600–610 nm. This allows label-free colorimetric readout for virus detection. Cartoon adapted from [42].

Influenza hemagglutinin (HA) surface protein binds to sialic acid glycan residues (α-2,6 and α-2,3 sialic acids) on the surface of human and bird cells. HAs expressed by AIV bind specifically to the α-2,3 sialic acid which is preferentially expressed in the intestine of water-flow, while HA proteins from human-adapted viruses prefer to bind to the α-2,6 sialic acid glycan, mainly expressed on the epithelial cells of the human upper respiratory tract. Based on this difference in sialic acid linkages, biosensors were developed for detecting and differentiating between avian and human influenza viruses. Glycan-immobilized field effect transistor biosensor was shown to detect and discriminate between human (H1) and avian (H5) influenza viruses at attomolar-level sensitivity [37]. Surface plasmon resonance [38, 39], optical waveguides [40] and quartz crystal microbalance [41] were also employed in glycan-based AIV detection. HA binding to sialic acid attached to gold nanoparticles allows detection of influenza virus in solution without any pretreatment or amplification step [42]. This reaction may produce a signal in colorimetric test that is linearly proportional to the virus titer (Figure 2). A recent study reported that glycan can be printed onto glass slides to generate microarray [43]. The microarray was shown to capture different strains of influenza virus with a clinical relevant limit of detection (ten plaque forming units) allowing virus diagnostics.

**Figure 3:**
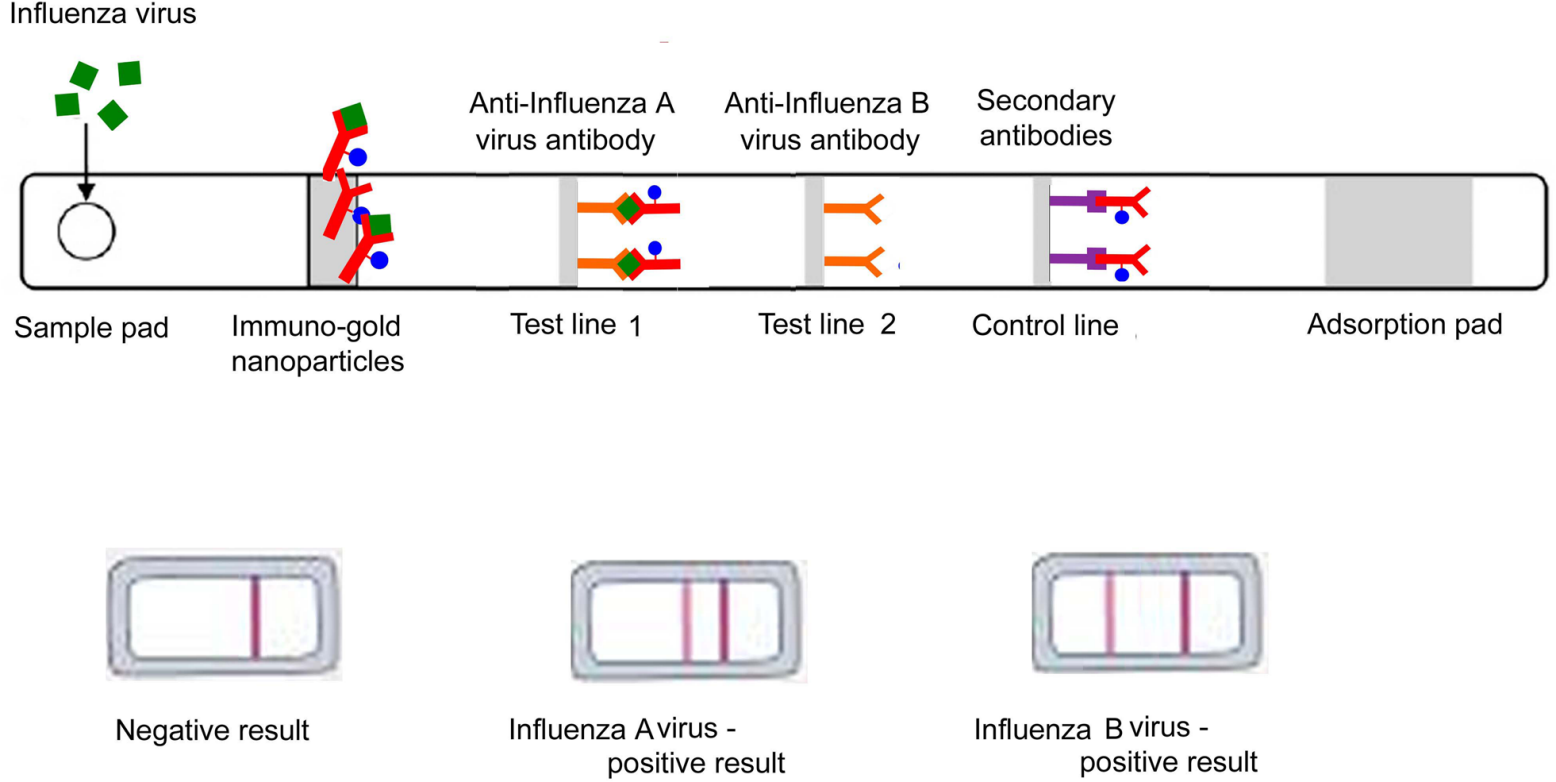
**Lateral flow strips for detection of influenza A and B viruses.** Schematic presentations of a lateral flow tests realized on a nitrocellulose strips with immobilized antibodies against influenza virus A and B. A sample containing influenza virus flow by capillarity from the sample pad to bind test and control lines. In contrast, a sample without target virus flows from the sample application pad and binds only to the control line.

Most of portable antibody-based methods for influenza virus diagnosis, including those being commercialized, are lateral flow tests [44]. Lateral flow tests may detect a specific influenza biomarker in a complex media thanks to their chromatography component consisting of series of pads that transport samples spontaneously during the test (Figure 3).

Samples to be detected for influenza virus by lateral flow test are solubilized in a detergent containing solution and deposited on the sample pad. This step initiates lateral flow of sample components. In the first step of detection a specific influenza virus biomarker is recognized by antibodies carried by gold-nanoparticles pre-adsorbed on the conjugate pad. Usually antibodies raised against preserved epitopes in viral nucleoprotein are used for this step. Influenza viruses complexed with immune-gold nanoparticles reach the test lines. Two test lines with pre-immobilized antibodies that specifically recognize either influenza A or B virus bind to different epitopes of the virus and, in that way accumulate immune-gold nanoparticles carrying the viruses. The accumulation of gold nanoparticles results in an appearance of a visible red line. Usually, antibodies recognizing specific epitopes in HA proteins are used for the test line. Finally, non-bound immune-gold nanoparticles arrive at the control line which harbors the secondary antibody, showing the second visible red line. In the absence of viral particles in the sample, the immune-gold nanoparticles flow alone and bind only to the control line. Thus, two colored lines stand for positive result while a single colored line corresponds to negative result (Figure 3). In most cases antibodies can distinguish between influenza A and B viruses but still not able to differentiate within subtypes and, thus, between LPAIV and HPAIV stains. In contrast, aptamers generated against specific AIVs or specific RNA/DNA primers immobilized on the test lines allow AIV sub-typing.

The extreme simplicity to use, efficiency, reliability and label-free detection characterized lateral flow tests. However, their sensitivity is sufficient for detecting proteins but has to be improved to allow detection of viral particles in a complex medium as fecal swab sample. It was shown that silver nanoparticles added to the test line can amplify the colorimetric signal up to 1000-fold [45]. A limit of detection 0.09 ng/mL was estimated for AIV detection by lateral flow test amplified with quantum dots [46]. For comparison, this test showed 100-fold higher sensitivity than ELISA performed with the same antibodies. Recently, a quantum-dot based lateral-flow immunoassay system was proposed for quantitative detection of influenza A virus subtypes H5 and H9 [47]. The modification of specific antibodies with quantum-dot amplified signal and permitted a quantitative read-out of the virus detection under an ultraviolet lamp.

**Figure 4:**
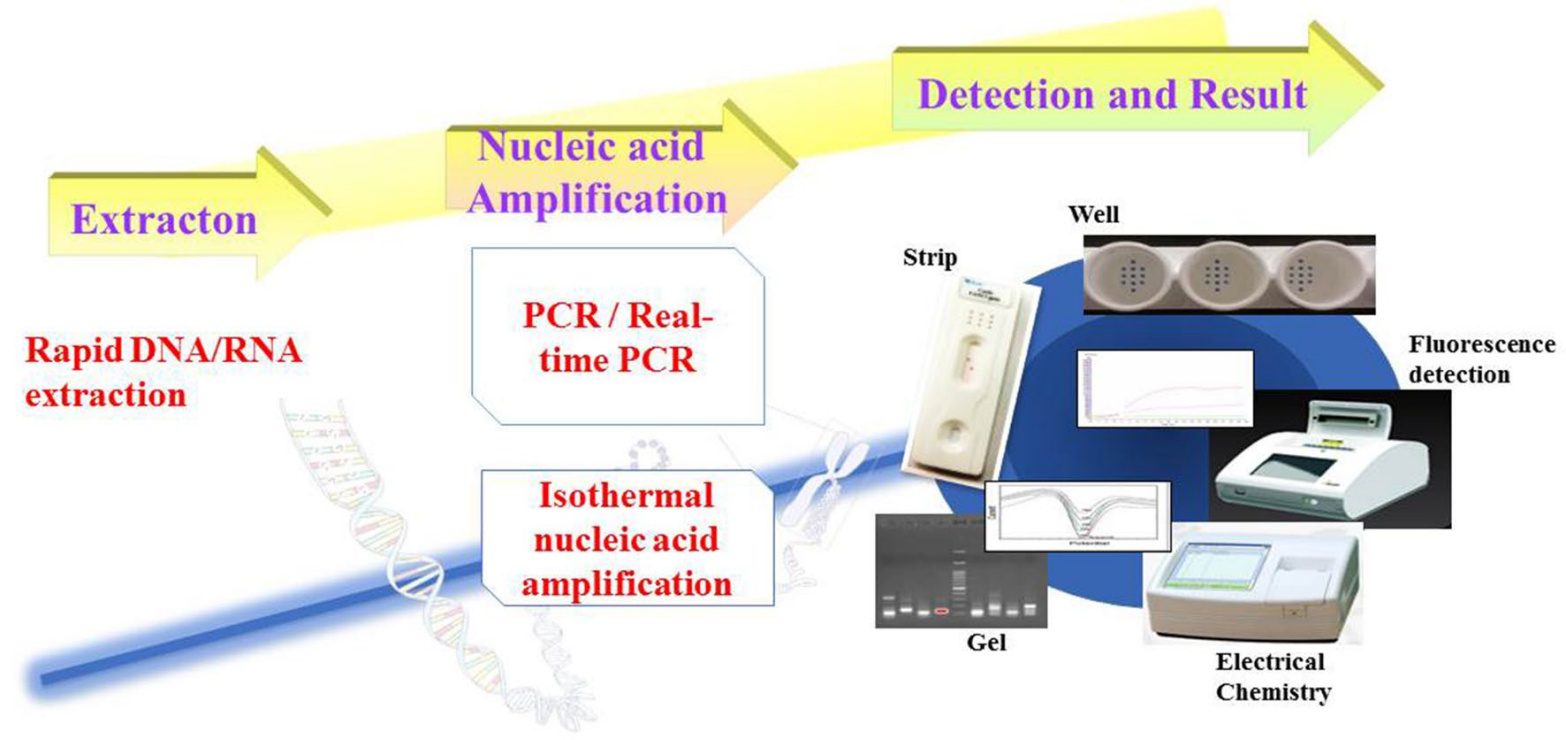
**Detection of influenza virus RNA.** Available combinations of portable nucleic acid extraction, amplification and detection methods for virus detection.

**Figure 5:**
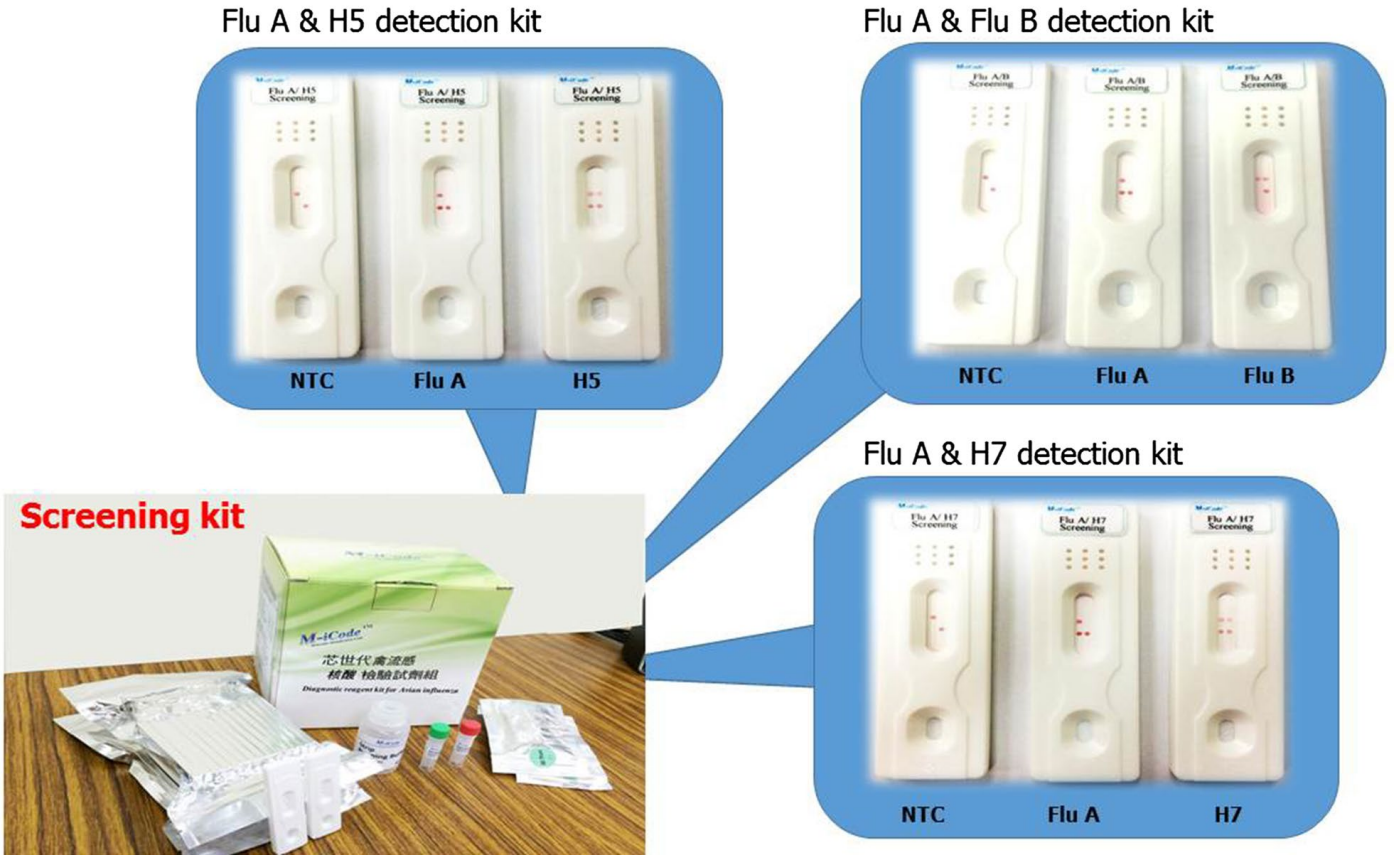
**Lateral flow kits for detection of influenza viruses.** RT-RPA with lateral flow Influenza virus detection kits for influenza A, influenza B, Influenza H5 and H7 subtypes. Pictures provided from Hawk Scientific Co., Ltd and GenProNex Biomedical INC. (Flu A: influenza A, Flu B: influenza B).

Several commercial PCR, RT-qPCR, RT-RPA kits and portable machines, in a mobile diagnostic suitcase, are available on the market for AIV detection and subtyping. Some of them combine lateral flow test after specific labeled primer-set amplification to increase the sensitivity of lateral flow system. It will be interesting to have efficient integrations of available and stable amplification and detection assays, for instance, the combination of PCR/isothermal amplification and biosensor technology. For instance, a portable and rapid assay for the detection of the emerging avian influenza A (H7N9) virus has been developed in a form of diagnostics suitcase. The test is based on reverse transcription recombinase polymerase amplification (RT-RPA) assay, isothermal amplification and a fluorescence detection machine. The workflow consisted of viral nucleic acid extraction, isothermal target nucleic acid fragment amplification and fluorescence detection [48]. Portable nucleic acid thermocyclers including PCR and isothermal amplification has become applicable for rapid on-site viral nucleic acid detection despite of the need of genetic materials isolation procedure. The combination of nucleic acid extraction, amplification and detection methods can be varied for different viral strain detection (Figure 4). RPA assays for AIV detection can be combined with lateral flow test. This combination provides a qualitative but not quantitative result (Figure 5).

Recently, an electrochemical immunosensor based on a specific anti-M1 antibody was shown to detect all serotypes of influenza A virus [49] with sensitivity similar to classical molecular methods (80–100 × 10^3^ PFU/mL). A lower effective limit of 1 × 10^3^ PFU/mL was achieved by coupling the anti-M1 monoclonal antibody to gold nanoparticles in a quartz crystal microbalance assay [50]. A sensitive plasmon-assisted fluoro-immunoassay was developed for the detection of the influenza virus by specific anti-M1 antibodies conjugated to gold nanoparticle-decorated carbon nanotubes. After influenza virus binding to these mixed nanoparticles, a fluorescent signal was produced by addition of cadmium telluride quantum dots. A photoluminescence intensity of quantum dots was shown to vary as a function of virus concentration, with a detection limit of 50 PFU/mL. In another study, it was demonstrated that an electrochemical immunosensor provides a very sensitive platform for detection and quantification of PB1-F2 protein of Influenza A virus in infected cells [51, 52]. The detection limit of the device was determined as 0.42 nM PB1-F2 [51]. A PCR-free paired surface plasma wave biosensor has been successfully developed for detection of 2009 pandemic influenza A virus [53]. The proposed diagnostic method is rapid, sensitive and accurate but only applicable for laboratory diagnostics.

**Figure 6:**
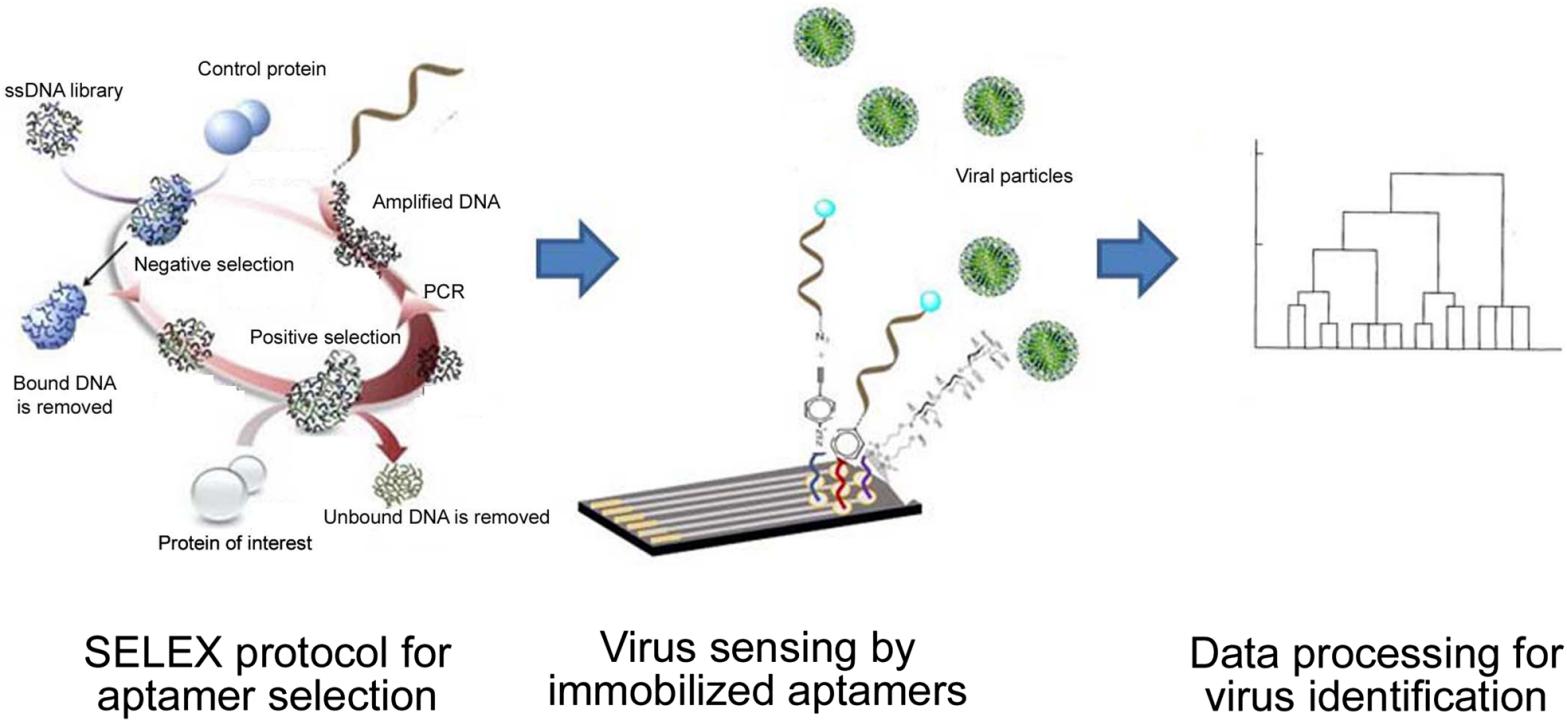
**Aptamer-based detection of influenza viruses.** Schematic representations of aptamer development and virus detection. Selex procedure is applied for selection of specific aptamers. These sensing elements are immobilized on the sensor surface to bind efficiently to the viral proteins in infected samples. The recognition signal is proceeded to provide diagnostic.

Artificial nucleic acids with defined 3D-structure, called aptamers that allow discrimination between different serotypes of influenza viruses are generated using systematic evolution of ligands by exponential enrichment (SELEX) technology (Figure 6). Aptamers are a good alternative to antibodies, because their production is not expensive and time-consuming and does not require animal hosts. Aptamers can present binding affinities in the picomolar range, thus much higher sensitivities than most antibodies can reached. Various aptamers generated against AIV proteins have been developed [54, 55]. These aptamers showed relatively strict specificity for the influenza virus with broad subtype specificities such as H5N1 and H1N1. For instance, Wang et al. [55] and Fu et al. [54] characterized aptamers that specifically bind to H5N1 but not to other AIV subtypes. Aptamers generated against avian influenza viruses attached to quantum dots allowed both detection of viral particles and their labeling for ultrastructural characterization of infected samples [56]. Interestingly, some aptamers bind to HA protein site that recognize sialic acids at the surface of a host cells. Consequently, they can attenuate virus infectivity which suggests their potential applications as anti-viral agents [57, 58].

Many groups develop multiplex detection of different influenza subtypes on a single device. Microarrays for diagnostic applications are elaborated either for monitoring pathogen virulence genes or for simultaneous screening of multiple pathogens. The majority of influenza microarray utilizes a panel of primers for multiplex PCR amplification of the HA, NA and M1 genes of influenza viruses. Li et al. [59] coupled a DNA microarray and multiplex reverse transcriptase PCR microarrays to provide subtyping of influenza virus strains. Similarly, Kessler et al. [60] developed a three-dimensional DNA flow-through biochip for typing and subtyping of influenza viruses. Wang et al. [61] designed a microarray-based detection and genotyping of a wide range of respiratory viruses. In one another study, Townsend et al. [62] described a microarray, for the relatively rapid identification of influenza A virus subtypes H1N1, H3N2 viruses circulating in the human population as well as the highly pathogenic avian A/H5N1 virus that was isolated from poultry in Southeast Asia and that spread to Europe. This test demanded extraction and amplification of the viral RNA, and thus cannot easily be used on-site. However, the device provided the absolutely correct types and subtypes for an average of 72% of the isolates. The authors emphasized that the failures were due to the nucleic acid amplification step rather than limitations in the microarray.

Although microarrays are typically used a fluorescent read-out there is a great potential of electrochemical microarrays for detection of influenza viruses. For instance, the microarray silicon chip for the detection of influenza A virus based on more than 12 500 electrodes, each carrying specific oligomers allowed the genotyping and identification of HA and NA influenza A proteins and virus subtyping [63]. Current developments are focused on miniaturization and automatization of microarray biosensors for building portable diagnostics platforms for avian influenza viruses.

## Detection of *Mycoplasma* and other bovine mastitis pathogens

Mycoplasmas are the smallest bacterial cells that lack a cell wall around their cell membrane, which makes them insensible to many common antibiotics. Antibiotics help preventing some clinical signs but cannot eliminate infection. Various mycoplasmae strains infect animals, but usually play a secondary role in infections by exacerbating pre-existing disease. Nevertheless, *Mycoplasma bovis* (*M. bovis*) can play a primary role of pneumonia, mastitis or arthritis in cattle. *M. bovis* is considered as one of the most pathogenic and the most frequent *Mycoplasma* species. It is estimated that *M. bovis* infection causes €144 million in the European cattle industry [64]. Methods used for diagnosis of *Mycoplasma* infection include bacterial culture, fluorescent antibody-based test, serological tests and PCR. Serological tests cannot be applied for early diagnosis or for detection of acute infection since serum antibody biomarkers rise at 10–14 days after acute infection. The somatic cell count as a reference method for monitoring milk quality allows mastitis diagnosis. This simple but labor-intensive assay implies methods as microscopic analysis or cytometry for raw milk. Both methods are slow and require well-trained staff to provide result accuracy and reproducibility. Early diagnosis is however of the extreme importance due to the high costs of treatments. Some portable somatic cell counters have been validated and are used as cow-side test for mastitis control at a dairy farm. Although they are very simple to apply, they have low sensitivity and specificity and usually cannot identify the pathogen strain [65, 66]. Especially, the early diagnosis of bovine mastitis is important regarding its huge impact on farm economics due to treatment cost and reduction in milk production.

Biosensors have been developed to detect specific *Mycoplasma* biomarkers in a rapid and easy-to-use diagnosis manner. Majority of in-field biosensors are developed to detect NAGase and haptoglobin biomarkers in milk samples. Expression of both bacterial proteins is characteristic for the acute phase inflammation. For instance, oxido/reduction process of 1-naphthol, which is a substrate of NAGase can be easily detected and quantified using carbon electrodes. Pamberton et al. [67] have reported an electrochemical sensor based on a screen-printed carbon electrode which detected NAGase protein in milk samples with a limit of detection of 10 mU/mL. Surface plasmon resonance was employed to monitor binding of haemoglobin to haptoglobin immobilized on the surface of the chip [68]. The formation of this protein complex resulted in changes in mass attached to the chip surface which indicates mastitis. Though, when this biomarker is targeted to test milk samples false positive results are obtained in some milk samples that contained blood traces.

A single-stranded DNA aptamer showing high affinity and specificity against P48 protein of *M. bovis* has been used in a competitive enzyme-linked aptamer assay for the detection of *M. bovis* in sera [69]. P48 protein is an optimal biomarker for *M. bovis* since it is an invariable protein that is localized on the membrane surface of *M. bovis*. A competitive enzyme-linked aptamer assay using the biotin-labeled aptamer of P48 protein was applied in an indirect diagnostic test. The sensitivity and selectivity of the test were similar to commercial ELISA kits.

Mastitis as an inflammatory infection may be caused not only by *Mycoplasma*, but by many other bacteria. The most frequent clinical infections in dairy cattle are caused by about ten different bacterial pathogens as *Staphylococcus aureus*, *Streptococcus agalactiae*, *S. bovis*, *S. canis*, *E. coli*. Consequently for controlling the disease spread and for targeting antimicrobial therapy a rapid strain identification and quantification of the microbial load in milk samples is needed. Nowadays, PCR or RT- PCR-based tests are employed for laboratory testing. As mentioned above, these methods are rapid and sensitive but their application for milk samples is sometimes impeded as milk contains calcium ions and proteinases that act as PCR inhibitors. Various procedures are established for DNA extraction and purification from the raw milk. Reagents for these additional steps are included in commercial kits which additionally increase reaction time and the price per analysis. Nevertheless some PCR-based kits allow identification of total bacterial strains causing mastitis and detection of their antibiotic resistant gens [70].

**Figure 7:**
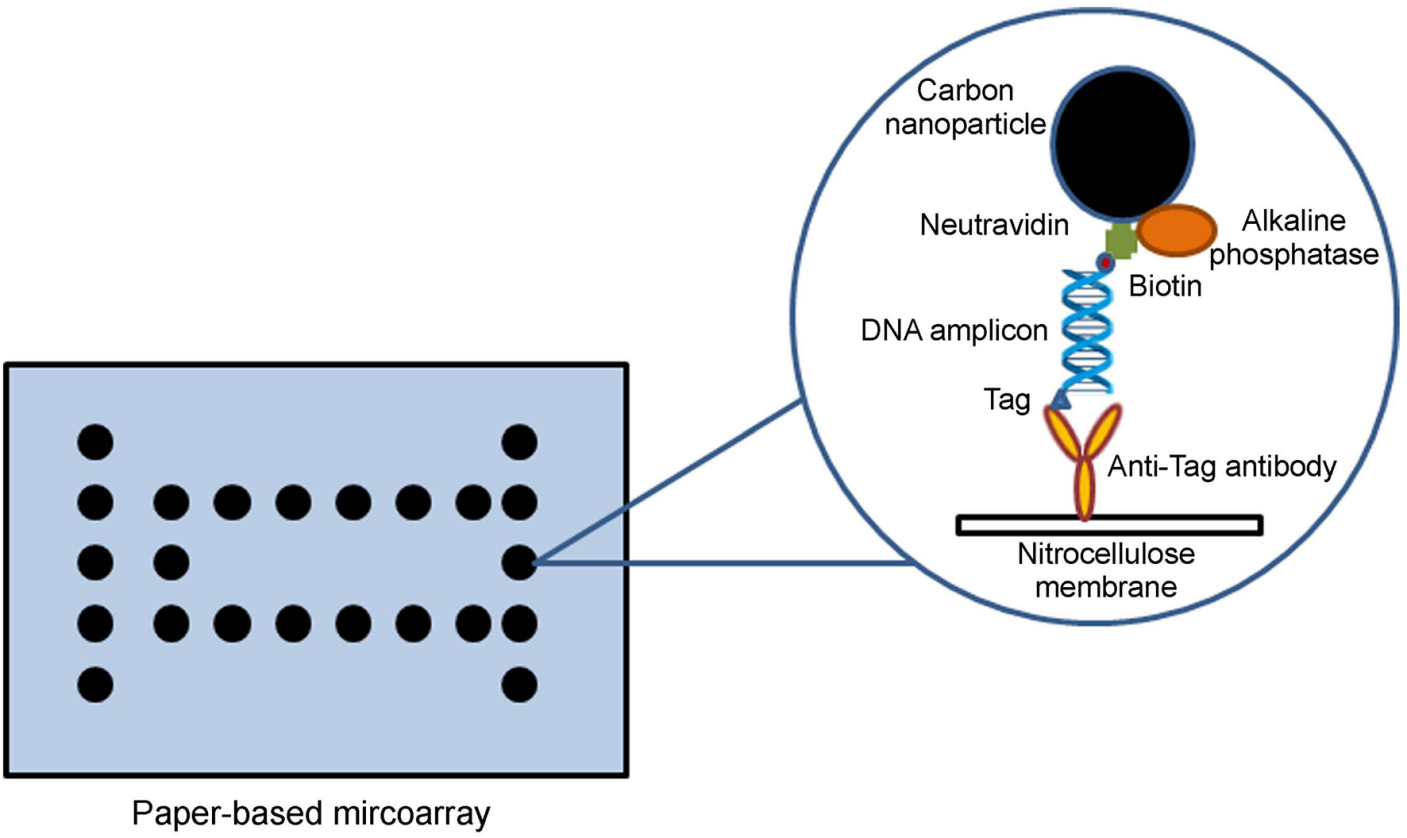
**Microarray for detection of bacterial nucleic acids.** Schematic representation of microarray for mastitis bacteria detection realized on porous nitrocellulose membrane slides. Illustration adapted from Mujawar et al. [73].

Advances in the development of the nucleic acid microarray have permitted automatization of the protocols and development of multiplex biochips for detection of various dairy pathogens [71]. For instance, a biochip based on DNA amplification of genes characteristic for mastitis causing pathogens was shown to efficiently detect six other pathogens in addition to *M. bovis* in bovine milk with a limit of detection of 10^3^ CFU/mL [72]. More recently a test that combined a rapid PCR with a nucleic acid microarray immunoassay was proposed [73]. This colorimetric test allowed simultaneous detection and identification of six strains from four different mastitis causing pathogens within less than 3 h. In the microarray a set of specific antibodies that recognize tags attached to specific PCR fragments were immobilized by printing onto nitrocellulose membrane. After PCR with tagged primers, the double-tagged amplicons were captured between the antibodies printed onto the nitrocellulose and carbon nanoparticles carrying alkaline–phosphatase. In the presence of the alkaline–phosphatase substrate, the black spots appear on the membrane that can be easily seen by the naked eye (Figure 7). The selectivity of the test was obtained by attaching different tags to primers specific for each pathogen of interest. This experimental approach may be integrated into a self-contained, simple and disposable cassette for point-of-care multi-pathogen molecular diagnostics.

An electrochemical sensor based on electrochemical impedance spectroscopy was developed for detection of pathogenic *Staphylococcus aureus* ATCC25923 bacteria by Braiek et al. [74]. A linear relationship between the increment in the electron transfer resistance and the logarithmic value of *S. aureus* concentration was observed between 10 and 10^6^ CFU/mL. The limit of detection was low as 10 CFU/mL, and the reproducibility was calculated to 8%. In addition, a good selectivity *versus E. coli* and *S. epidermidis* was demonstrated.

## Detection of *Clostridium perfringens*

*Clostridium perfringens* (*C. perfringens*) is a gram-positive, anaerobic, fermentative, spore-forming soil bacterium that may produce one or several exotoxins. At least 17 different *C. perfringens* toxins have been identified [75, 76], some of them being very potent causing animal and food-borne illnesses, and are even considered to be bioweapons. *C. perfringens* are classified into five isotypes (A, B, C, D and E) regarding five major toxins they produce [alpha, iota, alpha, beta (1 and 2), epsilon toxins, and enterotoxin E] [77]. In addition, novel toxins have been identified, as NetB, which was isolated from chickens with necrotic enteritis [78]. The toxin productions in animal gut are associated with specific enterotoxemias characterized by a variety of symptoms and traumatic infections. *C. perfringens* strains may infect birds, dogs, horses, pigs, lambs, cattle, sheep, goats and other domestic animals. Although *C. perfringens* is an important enteric pathogen for almost all domestic animals, the most advance studies on its pathogenicity have been made for broiler chicken. For instance, alpha- and beta-toxin of *C. perfringens* are responsible for clostridial enteric disease in poultry. This necrotic enteritis cause significant economic losses to the global poultry industry due to high mortality rates. Furthermore, some strains of *C. perfringens* that produce enterotoxins at sporulation are responsible for foodborne disease in humans.

The isolation of *C. perfringens* alone is not sufficient for diagnosis but has to be confirmed with histological evaluation of lesions. The lesions in the lamina propria associate with a strong inflammation is characteristic for early stages of infection, while in later stages, necrosis of mucosa and association of gram-positive rods with the lesions is characteristics [79]. The infections are usually treated by antibiotics. In farms that stopped using antibiotic growth promoters, outbreaks of clostridial infections increase dramatically. In consequence, the improving diagnostics and surveillance of *C. perfringens* are crucial.

Diagnosis of enteric diseases produced by *C. perfringens* is usually difficult since these bacteria can normally colonize bird gut. Moreover, it was shown that alpha-toxin of *C. perfringens* is not an essential virulence factor in the pathogenesis of necrotic enteritis in chickens. Thus, the main challenge is to determine the biological activity of *C. perfringens* in terms of rapid strain identification and differentiation of pathogenic from non-pathogenic strains. A solution can be found in cell-based detection systems which are emerging biosensor technologies that detect the biological activity of pathogens or toxins. Those biosensors have mammalian cells as sensing elements and allow monitoring perturbations in cell physiological activities following exposure [80]. Cell-based biosensors are capable of detecting the presence of pathogens or active toxin in clinical, environmental and food samples [2] rendering accurate estimation of the risk associated with the agent identification. Different device designs are proposed from a 96-well plate to modified electrodes carrying mammalian cells [80].

Each isotype of *C. perfringens* carries a defined subset of virulence genes coding for toxin-producing sequences. Sergeev et al. [81] immobilized specific oligoprobes onto a multipathogen oligonucleotide microarray to detect six toxin-producing sequences in *C. perfringens*. Sequences encoding the different toxins hybridized strongly and specifically to the corresponding oligoprobes. The microarray-based test was applied on fluorescently-labeled amplicons obtain by initial PCR amplification step [81–83]. After hybridization, the microarray was scanned to measure the presence or absence of signal above background as an indicator of bacterial presence in the tested sample. This experimental approach was used for simultaneous analysis for several bacterial stains, their toxin genes or drug resistance determinants on a single-chip platform [84].

Finally, toxins produced by *C. perfringens* may be detected using specific antibodies integrated onto an immunosensor. Such a device was demonstrated for sensitive and label-free detection of epsilon toxin produced by *C. perfringens* [85]. The sensor was obtained using an epsilon-toxin specific monoclonal antibody immobilized onto single walled carbon nanotubes. By controlling the morphology of the carbon nanotube assembly the sensor was adapted for detection of analytically relevant concentrations of toxin (nM range) and with sensitivities comparable to those of ELISA.

## Detection of bluetongue and epizootic hemorrhagic disease viruses

Bluetongue a major non-contagious infectious disease of domestic and wild ruminants (mainly sheep, cattle, deer) is caused by the bluetongue virus which is an *orbivirus* of the *Reoviridae* family. Bluetongue virus is transmitted by the bite of a female of certain species of midges of the insect family *Ceratopogonidae* but can also infect embryos via the placenta or be transmitted via seminal fluid and colostrum [86]. World Organisation for Animal Health (Office International des Epizooties, OIE) listed bluetongue virus because of its economic impact. The worldwide losses, estimated to 3 billion US$ a year [87], mainly affect ovine and bovine rearing industries. The estimated cost of bluetongue outbreaks in Scotland is £100 million per year (£30 million in direct losses and £70 million in indirect losses [88]), while the US losses in trade and associated testing of cattle for bluetongue virus status has been estimated at $130 million a year [89]. It is worth noting that although the mortality and morbidity of bluetongue in cattle are rare; cattle can be infected by the virus without showing any clinical sign and in that way acting as reservoirs and contributing to the virus transmission. In consequence, economic losses come not only from direct damages caused by death, or reduced meat and milk production, but also from indirect effects due to the restriction in animal, semen or serum exporting. The clinical manifestations of bluetongue disease range from an unapparent or mild disease, to pyrexia, tachypnea, lethargy and even fatal disease [90].

In contrast to bluetongue virus which mainly affects sheep, strains of epizootic hemorrhagic disease virus, another distinct species within the genus *Orbivirus*, may exhibit high mortality and morbidity in both sheep and cows. As both diseases have similar clinical symptoms, both can be transmitted by the same insect and both have significant negative impacts on trade, the diagnosis of *Orbiviruses* is important. Current methods for bluetongue and epizootic hemorrhagic disease virus include numerous nucleic acid amplification assays, genotyping viral isolates, DNA microarray and next-generation sequencing [91]. Promising advanced methods, such as fluorescent microsphere assays that can be adapted to single-tube multiplexing or multiple-well multiplexing, allow simultaneous detection of various viral RNA by in-solution hybridization [92]. These tests are currently under validation in some laboratories. However, other alternative amplification methods, as loop-mediated isothermal amplification which can allow pen-site testing, or lateral flow test have still not be applied for *Orbivirus* detection. Nevertheless, a rapid lateral flow test for the detection of bluetongue virus-specific antibodies has been commercialized and recently validated [93].

**Figure 8:**
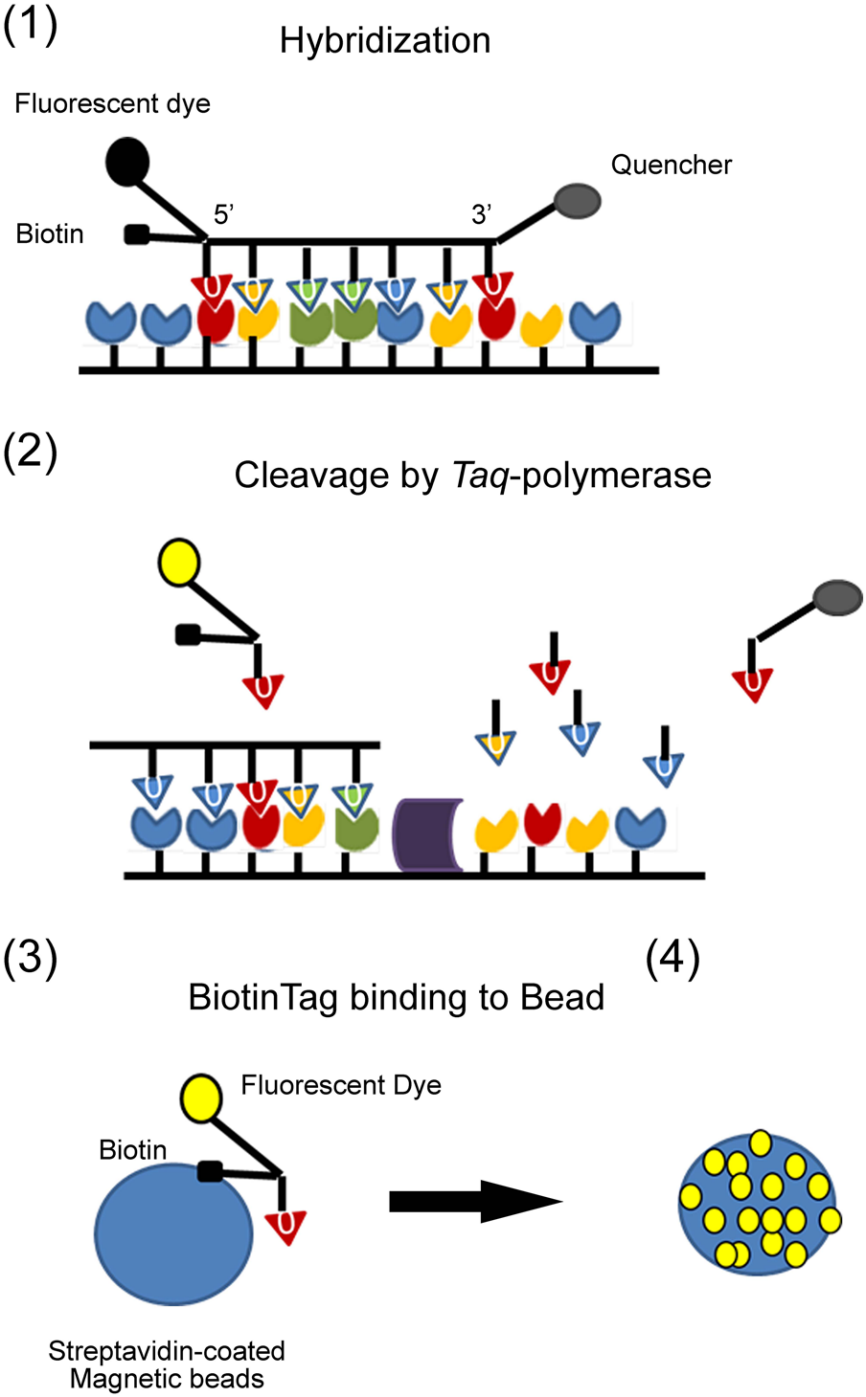
**Fluorescent detection of viral nucleic acids on magnetic beads.** Schematic presentation of the FRET-based magnetic biosensor for *Orbiviruses* detection. (1) A nucleic acid probe labeled with florescent dye, biotin and dark quencher hybridize with the complementary probe, (2) the fluorescent dye is separate from the quencher in each PCR cycle, and starts to produce light, (3) the fluorescent dye bind to streptavidin-coated magnetic beads via biotin tag, (4) about 1000 fluorescent-labeled probes bind to a single beads giving an increased fluorescent signal. Cartoon adapted from [94].

Danielli et al. [94] reported a rapid detection of Ibaraki virus causing epizootic hemorrhagic disease at picomolar concentrations by magnetic modulation and synchronous detection. This assay based on fluorescent-labeled oligonucleotide detection in a homogeneous solution can be integrated into the portable device and use for in field rapid virus diagnosis. The cDNA of the nonstructural NS3 protein expressed by the Ibraki virus served as a biomarker. The complementary nucleic acid probe was labeled with three different tags: a fluorescent dye and biotin were attached as a double-tag at the 5′ end, while a dark quencher was attached at the 3′ end (Figure 8). Following a PCR cycle, the probe was attached to the streptavidin-coated magnetic bead via the biotin tag. It was shown that each magnetic bead may attach thousands of labeled probed, which allowed to concentrate probes into the detecting area. Sample concentration and separation can be easily performed by external magnetic field. In this assay, during PCR, the fluorescent energy transfer (FRET)-based probe hybridized with its complementary sequence (Figure 8). Then, FRET-based probe is cleaved by *Taq*-polymerase which allows the fluorescent light to be produced. The intensity of the fluorescent light can be calibrated to the pathogen concentration, and used as measure of viral titer in infected samples. In comparison, authors shown that their system distinguish target from control probe after a single cycle of PCR while classical RT-PCR gave significant signal only after 12 cycles, while laser scanning microscopy required 18 amplification steps to give signal above the threshold level. This promising biosensor was shown to detect 1.9 pM of the virus NS3 cDNA within 18 min, without needing any separation or washing step.

## Detection of *Eimeria* species

Intracellular protozoan parasite *Eimeria* causes coccidiosis which represents an important health problem in the poultry industry worldwide. The economic losses of the poultry industry due to coccidiosis are estimated to US$7 billion a year, mostly due to the late diagnosis because symptoms are not visible at the initial stages of infection. *Eimeria* undergoes rapidly its life cycle inside cells in intestinal tract of the infected bird. Upon finishing the internal phase in which the parasite is multiplied and excreted as oocysts in the faeces, it undergoes sporulation in the external phase and becomes infective. Infection in chicken leads to low feed conversion rate, reduced weight gain and increased mortality. At least seven different *Eimeria* species can affect poultry showing no cross-immunity between them [95]. Usually under field conditions, coccidiosis is caused by infection with mixed but one dominant *Eimeria* specie. The damage occurring in the intestinal tract may facilitate secondary infection by some nonrelated bacteria, as documented for *C. perfringens* [96], or *Salmonella* Typhimurium [97, 98].

Traditional diagnosis employed in surveillance and control of coccidiosis is based on correlation of parasite size, morphology and site of infection with histological observation of lesions in infected birds. In addition to be complex and time consuming, these methods may also confuse coccidiosis with histomoniasis and salmonellosis due to their similar lesions. Biochemical and molecular diagnostic tools involve DNA based high-throughput analysis which permits to distinguish between distinct genetic parasites [99]. New multiplex PCR assays, using primer pairs characteristic for all seven *Eimeria* causing infections in poultry, are performed on oocysts purified from the feces [100]. Application of PCR-based assay for the presence of *Eimeria* oocysts on farms indicated limitations because of the assay low sensitivity (a minimum detection level was found to be 20 oocysts). This points out that classical PCR analysis cannot be applied for early infection diagnosis [101]. Advances PRC technologies provide methods more adapted for early diagnosis as random amplification of polymorphic DNA PCR, DNA fingerprinting protocols and qPCR [102–104].

An alternative to classical PCR is a loop-mediated isothermal amplification (LAMP) method reported for the first time by Notomi et al. [105]. LAMP is highly sensitive, specific and simple nucleic acid amplification method that functions at single constant temperature. It is also a time-saving method since an entire amplification is reached within less than 60 min. Overall, LAMP is quite adapted for elaboration of pathogens point-of-care diagnostic kits. LAMP is allowed by autocycling strand-displacement reaction using a set of oligonucleotides specific for different DNA sequences within the target genomic region and formation of a loop-structured amplicon. To improve amplification, additional loop primers may be added to the reaction. Barkway et al. [106, 107] have developed a panel of sensitive LAMP-based assays for diagnosis of seven *Eimeria* species that infect chickens. The test validation was performed on DNA extracted from mechanically disrupted *Eimeria* oocysts purified from faecal material. This study suggested that, although non-quantitative, LAMP-based diagnostics of coccidiosis parasite show many advantages: (i) the colorimetric read-out provided an instant result as no requirement for electrophoresis step; (ii) test sensitivity allowed parasite detection upon ongoing infection, and at early stages when any visible lesions can be resolved; (iii) LAMP was less sensitive to PCR inhibitors present in tested samples as some metal-ions or proteases compared to PCR and, finally, (iv) the completed test was cheaper than ~£1 per sample. Thus, simplicity, low cost and no requirement for sophisticated laboratory equipment make the LAMP-colorimetric paper test a good candidate for in field applications.

## Detection of foot-and-mouth disease viruses

Foot-and-mouth disease virus causes an extremely infectious and contagious disease of cloven-hoofed animals as cattle, pigs, sheep and many wild species. Although showing a low mortality, foot-and-mouth disease has the global impact estimated between US$6.5 and 21 billion a year, due to high morbidity of the disease and the huge numbers of animals affected [108]. It was estimated that one infected cow can infect more than 70 cattle, which makes foot-and-mouth virus the most infectious human and animal pathogen known. The clinical symptoms of food-and-mouth disease are formation of painful fluid-filled vesicles (blisters) or some erosion on the mouth tissues as tongue and lips, or other parts of the body where the skin is thin, like between the two toes of the feet. The pain causes many supplementary symptoms as depression, excessive salivation, lameness, anorexia and reluctance to move or stand.

The developed countries have eradicated the foot-and-mouth disease but widespread and long distance movements of animals sometimes re-incur virus. The outbreaks in disease free regions cause economic losses of about US$1.5 billion a year. Therefore a rapid implementation of the measures and a coordination between regions and countries are needed to control disease spread [109]. However, even nowadays the virus identification cannot be done in all endemic regions. Many developing countries during the suspected outbreaks have to send infected samples to international laboratories because of the lack of resource and laboratory equipment. The transportation represents a major biosecurity risk, increases time of analysis and risk of sample degradation and has additional cost. In consequence, there is a need for a portable device for in field foot-and-mouth diagnosis to assure better disease control, surveillance and management in outbreaks.

The food-and-mouth virus belongs to the *Aphthovirus* genus of the *Picornaviridae* family. It consists of a single-stained positively-charged RNA genome surrounded by a capsid composed of four structural peptides VP1-4. There are seven immunologically and genetically distinct serotypes. There is no cross protection between different serotypes which implies that an outbreak diagnostic of both virus and of their serotype have to be done. Classical methods for the food-and-mouth diagnostics involve virus isolation, ELISA and RT-PCR tests [109]. Recently an improved duplex one-step RT-PCR assays was validated [110]. The main advantage of this test is in the co-amplification of foot-and-mouth virus RNA and host β-actin mRNA. Host mRNA served as an intern control to ensure accuracy and to avoid false negative results that occur when quality of RNA in samples is poor. Although the assay was validated as specific and sensitive, it is only adapted to laboratory diagnostics.

Fowler et al. [111] reported a lateral flow device for recovery of food-to-mouth viral RNA. In their assay the virus detection and subtype identification were performed in laboratory facilities while the lateral flow device was only proposed to improve field sample preservation during transport. To test the proof of concept, the authors applied epithelial suspension or cell culture infected with a food-to-mouth virus representing various serotypes on the antigen lateral flow strip. Specific antigen–antibody reaction resulted in development of the test line. This lateral flow device was then employed as a dry, thermos-stable and non-hazardous transport system of infected sample for subsequent nucleic acid amplification, sequencing or virus recovery in laboratory facilities.

Reid et al. [112] developed a rapid and simple chromatographic strip foot-and-mouth diagnostic test for field application. This device was shown to detect specifically foot-and-mouth disease virus antigen in laboratory and field condition. The chromatographic strip test contained a specific monoclonal antibody with broad reactivity for foot-and-mouth disease viruses. The device was validated with laboratory-based tests on a range of samples as nasal swabs, epithelial suspensions and probangs from clinical samples or from animals infected experimentally as well as in supernatant fluids resulting from their passage in cell culture.

Another example of a preliminary validated point-of-care test for foot-and-month disease diagnosis is a device that coupled reverse transcription loop-mediated isothermal amplification (RT-LAMP) with a lateral flow strips [113]. The test provided diagnosis within less than 1 h with no requirement for instrumentation because the test line is visible by a naked eye. Moreover, the test can be performed in a standard water bath or heating block because RT-LAMP technique does not require a thermocycler as it amplifies specific nucleotide sequences at a constant temperature. The validation of the device has shown a successful detection of foot-and-month disease virus RNA from epithelial suspensions without the need for prior RNA extraction. All these advantages together with a possibility that a RT-LAMP method can be adapted for a high throughput system make this assay attractive for field use.

## Detection of *Campylobacter*

*Campylobacter* is a Gram-negative spiral-shaped bacterium, mobile with flagella, which belongs to the *Campylobacteriaceae* family. It is microaerophilic and thermophilic microorganism that can grow well at the temperature range between 37 and 42 °C. *Campylobacter* can cause diseases in humans and animals including wild animals, pets and livestock species. *C. jejuni*, *C. coli* and *C. fetus* strains have been found worldwide. Particularly, *C. jejuni* and *C. coli* have been found in sheep, cattle, chickens, turkeys, dogs, cats and pigs while *C. fetus* has been found mostly in sheep, goats and cattle. *C. jejuni*, mostly found in poultry, may colonize the intestine of turkeys, chickens and waterfowl. Clinical signs of infection are enteritis with diarrhea, vomiting and fever, but also abortions and infertility, bovine genital campylobacteriosis, ileitis in hamsters, colitis in ferrets, enteritis in porcine. Lesions of infected young animals include edema of the mucosa of the ileum and cecum, mononuclear infiltration of the submucosa, villous atrophy with intraluminal accumulation of mucus. In addition, animals can be infected asymptomatically with any of *Campylobacter* strains. For instance, *C. jejuni* is not considered to be pathogenic in birds. Still, some cases of *C. jejuni* infection in chickens have been reported to cause enteritis and death.

Poultry is considered as the main source of human campylobacteriosis [114, 115], the disease caused by the infection with *Campylobacter*. The WHO recommended a control and surveillance of *Campylobacter* in poultry to reduce the risk for humans since this bacterium has a significant impact on public health. In fact, contaminated, undercooked poultry is responsible for 50–80% of cases of campylobacteriosis investigated. Nevertheless, contaminated beef and pork products are also responsible for some infections of people. Costs due to *C. jejuni* infections are estimated between US$1.5 billion to US$8.0 billion a year and about €2.4 billion a year, in the United States and Europe, respectively [116, 117].

The recommended procedure for detection of *Campylobacter* requires 4 days to provide response on its presence or absence, while up to 7 more days are required for *Campylobacter* strain identification. Microaerophilic conditions, a specific temperature, and selective enrichment media are required to grow *Campylobacter* in laboratory. However, classical plate based methods are not quite suitable for diagnostics *Campylobacter*. First, the strain differentiation between *C. jejuni* and *C. coli* is difficult using conventional cellular or biochemical methods, because of the similar characteristics of the two species [118]. Second, transport can stress bacteria making them viable but-not culturable on selective agar plates, thus making plate count methods inefficient.

Molecular biology methods, that use nucleic acids as target for the tests allow detection of the viable but-not culturable microorganisms, reduce the time required to obtain results and improve specificity. Various kinds of specific and sensitive PCR-based tests have been developed that are usually run in laboratories. Thus, they require the transportation of the samples from the site of analysis to the equipped facilities. Regarding the sensitivity of *Campylobacter* to a variety of environmental conditions, the transport should be fast to reduce the loss of viability.

A portable device could help farmers to obtain a rapid detection of the pathogen, reducing the spread of the microorganism in the livestock and consequently of the food that could cause human illness. Barletta et al. [119] proposed a test based on melting-point curve analysis which identifies post-PCR products of *C. jejuni*. They standardized a classical PCR test with primers already reported against 16S rRNA of the *Campylobacter* spp. The test can be run using a multiple PCR for performing detection of *Salmonella*, *Shigella* and *Campylobacter* spp. Although selective, the method requires agarose gel migration of samples, making it slow and not adapted for screening high number of samples. In contrast, fluorescence-based RT-PCR methods are more robust and faster because no post-PCR protocols are required. Unfortunately, both protocols require the extraction of DNA and, thus, depend on the efficiency of the enzymatic reaction. As results may depend on the reagents used for enzymatic reaction, PCR-methods avoiding the amplification step would be more accurate.

**Figure 9:**
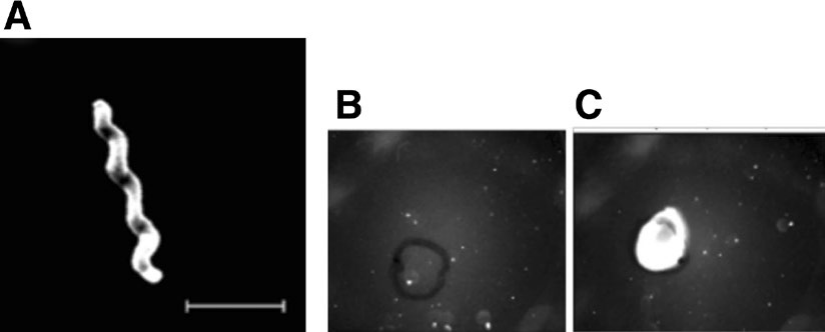
**Visualization and detection of** ***Campylobacter***. **A** Transmission electron microscopy of *Campylobacter* cell. Bar, 2 µm. **B** OLED biosensor probing of a negative control sample containing a non-*Campylobacter* DNA at 12.5 ng/µL, using a probe at 50 ng/µL. The spot was obtained upon deposition of 1 µL of the sample on the sensor surface. **C** OLED biosensor detection of *Campylobacter* DNA sequence at 12.5 ng/µL, using 50 ng/µL of the probe. The spot was obtained with 1 µL of the sample as previously explained [125].

In last 10 years various kind of sensors have been developed to improve the rapidity, specificity and simplicity of *Campylobacter* detection. For this, proteins, nucleic acids, aptamers, antibodies and whole cells have been used as bioreceptors for the development of specific point-of-care devices. Among them DNA-biosensors seem to be the most attractive diagnostic tool for their rapidity, specificity and cost effectiveness. Indeed, DNA-biosensors allow rapid, real-time monitoring of hybridization with the target nucleic acids. Specificity of the system relies on oligonucleotide probes covalently immobilized on the sensing surface. Techniques, such as optical [120], acoustic [121], electrochemical [122], microwire [123] and localized surface plasmon resonance [124], have been proposed for traducing the hybridization with the specific target nucleic acid to the pathogen detection. For instance, an organic light emitting diode (OLED) biosensor can be employed for the detection of *Campylobacter* in poultry meat samples, using a DNA probe attached to a glass slide (Figure 9). The labelling of the secondary DNA probe with an Alexa Fluor fluorophore allowed reaching a sensitivity of 0.37 ng/μL DNA and 1.5 × 10^1^ CFU/g of *Campylobacter* [125]. The method was demonstrated to be high sensitive even when no preliminary nucleic acid enrichment was performed. In addition, it is robustness, as zero false positive or false negative response have been obtained.

Zhang et al. [126] described the utilization of magneto strictive particle coated with three layers of silica to bind anti-*C. jejuni* antibodies. Such functionalized magnetic particles were shown to detect *C. jejuni* in water released from chicken factories. Although bacterial cells are typically diluted in water, the easy manipulation of magnetic particles allowed bacterial cells concentration giving a sensitive detection with the detection limit of 10^2^ CFU/mL. In another study, Wei et al. [127] proposed a biosensor based on surface plasmon resonance for identification of *C. jejuni*. Using specific antibodies against *C. jejuni* a sensitivity of 10^3^ CFU/mL was reached.

Gnanaprakasa et al. [128] developed an optical biosensor for the detection of *C. jejuni* based on diffraction optic technology and surface plasmon resonance. The hippuricase gene (hipO) of *C. jejuni* was used as a target sequence. After being thiolated the complementary probe was immobilized on the gold surface of the sensorchip. On-chip hybridization was traduced to an optical signal which allowed *C. jejuni* quantification in infected samples with a detection limit of 5.0 nM.

Wald et al. [129] used a lateral flow method to detect *C. jejuni* or *C. coli* reaching a sensitivity of about 7.3 log of CFU/g. Though not highly sensitive this device is portable and can be adapted to multiplex detection. Possibility to have multi-pathogen in field diagnosis will significantly reduce time of detection allowing the activation of control measures in a short time. The improving of the hygiene of poultry will reduce the number of infections caused by *Campylobacter* in farms and thus the costs of human hospitalization.

## Detection of *Salmonella*

*Salmonella* is a Gram-negative, rod-shaped, facultative anaerobic, non-spore-forming, motile enterobacteria, that can grow over the temperature range from 7 to 48 °C, and at optimum pH from 6.5 to 7.5. *Salmonella* is a cause of foodborne illness worldwide and is responsible of livestock infections that can be transmitted from animals to humans. Poultry are considered as one of the most important *Salmonella* reservoirs. Various serotypes of *Salmonella* overlap between farm animals and humans [130]. Animals are mainly infected through feed, drinking water or environmental sources. Poultry farmers which suffers flock losses due to *Salmonella* infection, suffers also for eggs and other consumable poultry products losses. Various different strains of *Salmonella* have been identified although only two species have been recognized: *S. enterica* and *S. bongori*. *Salmonella enterica* can be subdivided in subspecies, *Salmonella enterica* subsp. *enterica* (I), *Salmonella enterica* subsp. *salamae* (II), *Salmonella enterica* subsp. *arizonae (*IIIa), *Salmonella enterica* subsp. *diarizonae* (IIIb), *Salmonella enterica* subsp. *houtenae* (IV), *Salmonella enterica* subsp. *houtenae* (VI). *Salmonella bongori* was originally designated as *S. enterica* subspecies V. The most important subspecies involved in poultry infections are *Salmonella* Typhimurium, *Salmonella* Enteritidis, *Salmonella* Virchow, *Salmonella* Hadar. Among all strains, *S.* Enteritidis is the serovar mostly implicated in salmonellosis worldwide. Affected birds are anorectic, weak, have diarrhea and may die shortly after hatching. Those who survive rest small in size and frequently carry localized infection of the ovary. In consequence survived hens may transmit the infection to the eggs and produce infected chicks.

The ISO 6579:2002 (AnnexD) method is currently the EU standard method for diagnosis of *Salmonella* in poultry samples. Other standard culture-based methods are available. Some of them have been validated by other regulatory agencies. Most of these conventional culture-based methods comprise the following steps: (i) liquid pre-enrichment on non-selective medium followed by that on (ii) liquid selective media containing inhibiting compounds towards non-*Salmonella* bacteria, (iii) culture on solid selective agar, and (iv) isolation of suspected colonies from the solid media, their incubation in a specific solid media followed by additional biochemical and serological tests for strain identification. All culture-based methods need complex laboratory equipment and at least 1 week to give results. Moreover, some serotypes cannot be detected on the selective media used for isolation, thus, false negatives can be provided by the test. For instance, *Salmonella* Virchow cannot be cultivated on agar.

In consequence, immunological and serological tests have been developed for *Salmonella* diagnosis. The identification step that uses biochemical assays detecting a specific biomarker has reduced the volume of the reagents allowing the simultaneous inoculum of many wells on the same strip leading to a reaction that can give the answer within 24 h. Many of such kits are commercially available (as API 20E, bioMerieux; Enterotube II, BD diagnostics, etc.). Nevertheless, it is difficult to incorporate immunoassays into an array to detect multiple targets due to the high cross-reactivity between antibodies. On the other side, ELISA, although high sensitive and fast, still can give false negative results, cross-reactivity problems, and need a pre-enrichment step [131].

The utilization of molecular biology and PCR-based methods, such RT-PCR and Digital-PCR, can reduce the time for *Salmonella* detection to several hours. These methods may be employed even when serotyping fails due to the change or loss of the surface antigens. Various kinds of rapid tests for *Salmonella* have been developed and approved, including the VIDAS *Salmonella* (SLM) method, the immuno-concentration *Salmonella* (ICS) method, the Tecra Unique *Salmonella* test, and the polymerase chain reaction PCR based BAX™ system, approved by the Association of Official Analytical Chemists (AOAC) International. The results are obtained within 24 h. Also Dynal BeadRetriever, Pathatrix, and BioVeris M1M Platform based on immunomagnetic separation (IMS) systems, and many others are currently used. Gene-Trak^®^ (Neogen Corporation) bases on DNA probe hybridization [132] allows after the lysis of the target cells, the release of DNA for hybridization to the probe. Most molecular tests use an enrichment step to increase the sensitivity extending the time of the test and require supplementary equipment for the analysis.

In addition to food-borne pathogens detection and sanitary control of farms, the possible utilization of *Salmonella* for bioterrorism led to the development of portable biosensor-based detection methods. Biosensors may detect specific *Salmonella* proteins or DNA sequences in a rapid, specific, simple and sensitive way. Chai et al. [133] described a wireless magneto elastic mass-sensitive biosensor for the detection of *S.* Typhimurium, able to detect 1.6 × 10^2^ CFU/mL. Wang et al. [133] reported the utilization of microcantilever decorated with peptides having high affinities to various strains of *Salmonella* able to detect only one cell of the pathogen in an infected sample. The cantilever bends lightly, due to the capture of a bacterium by the specific peptide, and a laser activates the alarm. The authors used phage-derived peptides to obtain an array for parallel detecting of eight serovars. Phage display uses genetic modification of viruses that infect bacteria, bacteriophages, to insert a sequence of the peptide that specifically recognizes one biomarker into a phage coat protein gene. In that way, the phage starts to express the peptide on its surface. The displaying phages can be attached on the biosensor surface and assure specific *Salmonella* detection.

A lateral flow system for *Salmonella* AD, produced by the DuPont, uses two-step enrichment (16–22 h plus 6–8 h) before the sample insertion on the strip. Strips contained colloidal gold conjugated anti-*Salmonella* antibodies coated onto the sample pad. The test provides results in 10 min. Strips have been validated for *Salmonella* detection on raw meat, chicken sera, eggs, processed meat, fruits, vegetables, etc. This method was also confirmed on meat samples spiked with 1–4 CFU/25 g before the enrichment.

Bulut [134] reported the development of a lateral flow immunochromatographic test platform for *Salmonella* using the *invA* gene as a target. This gene encodes the protein responsible for the invasivity of the pathogen. The platform requires no labeling of the target as it detects amplicons obtained by using specific primers followed by sandwich hybridization. Nevertheless, the method is not quantitative since it involves a PCR step. Fang et al. [135] developed a lateral flow biosensor based on aptamers to detect *Salmonella*. This method needed an amplification step to produce an amplified ssDNA before its deposition onto the sensor membrane. The reported sensitivity was 10^1^ CFU/mL.

Microfluidic devices have also been used for the detection of *Salmonella*. Kim et al. [136] detected *S.* Typhimurium using a microfluidic device and specific anti-*Salmonella* antibodies able to efficiently capture *Salmonella* cells. *S.* Typhimurium was used to spike samples at various concentrations and establish the detection limit. A portable fluorometer using a LED unit detected the signal produced by the quantum dots conjugated to antibodies. The detection limit obtained for the sensor was 10^3^ CFU/mL *Salmonella* in chicken meat extract.

## Detection of bovine respiratory syncytial viruses

Bovine respiratory syncytial virus (BRSV) is spread worldwide and represents a major contributor of respiratory disease in cattle, especially in young beef and dairy cattle. The high prevalence of seropositive cattle infection is common in the cattle population making BRSV the most economically important and outstanding welfare issues for industrialized beef cattle producers and animal health organizations. The virus seems to spread by an aerosol route and can be easily transmitted by contact with respiratory secretions from infected cattle. Frequently, BRSV infection is associated with secondary bacterial infections that are treated by the massive use of antibiotics. In consequence, BRSV represents an additional public health-concern through the risk of antibiotic-resistance developing.

BRSV is a single-strained, negative-sense RNA enveloped virus classified as a Pneumovirus of *Paramyxoviridae* family. The genome of BRSV encodes for 11 proteins: nucleocapsid (N), phosphoprotein, large polymerase, transcriptional antitermination factor M2-1 and RNA regulatory protein M2-2 that all associate the genomic RNA, then attachment (G), fusion (F) and small hydrophobic protein that are transmembrane surface glycoproteins, while matrix or membrane protein (M) is a nonglycosylated and associates with the inner face of the envelope. In addition, two nonstructural proteins, named NS1 and NS2, accumulate within the infected cells.

After the incubation period estimated to take between 2 and 5 days, BRSV infection is usually limited to the upper respiratory tract giving clinical signs that may range from minimal to severe with dyspnea and death. Affected calves can have tachypnea, ocular serous secretions, dry muzzle, reduced activity, anorexia, and high fevers. The clinical signs may last until 7–10 days post-infection. Interestingly, the antibody response is frequently developed few days before clinical signs appear. Thus, testing serum samples pooled from a number of animals in a respiratory outbreak may help diagnosis. Still, although clinical signs may suggest BRSV infection, diagnosis of BRSV requires a laboratory confirmation.

PCR is commonly used as a molecular method for BRSV diagnosis. However, the virus isolation is difficult, except in some cases upon the acute phases of infection. Detection of negative-sense RNA genome or mRNA of the virus is even more delicate than detection of viral proteins. First, RNAs are sensitive to RNAse digestion and degradation and have shorter life time than proteins. Second, RSV mRNA is expressed cyclically, while protein expression is stable and increases in a regular manner over time. In consequence, ELISA test that quantify the RSV fusion protein subunits F0 and F1, expressed by all RSV strains, provides an efficient diagnostic tool. Similarly, the detection of specific antibodies in serum samples has proved useful to establish a diagnosis of BRSV antigen in the acute sample. Interestingly, the antibody titer in animals with developed clinical signs of the disease is usually higher than in the sample taken several weeks later.

Cai et al. [137] proposed a label-free electrochemical biosensor based on molecular beacon for detection of target mRNA from RSV. Molecular beacons are oligonucleotide hybridization probes in a form of a hairpin shaped molecules. Within the hairpin structure a streptavidin binding aptamer sequence was blocked for this assay. Upon hybridization of the beacon with specific nucleic acid sequence, the hairpin opens to allow binding to streptavidin-HRP protein complex. In that way, the hybridization event can be quantified by the enzymatic reaction using a HRP substrate, tetramethylbenzidine (TMB). The sensor was shown to detect 13 amol of RSV mRNA in a 4 µL total volume of a complex biological media. The high specificity and selectivity of the device was demonstrated as sensor detected specific RSV mRNA but not one base mismatched sequence.

In another study, the HRP-TMB reaction was used for electrochemical detection of RSV specific antigens [138]. For this, the polystyrene array slide that allows antibody immobilization was attached with disposable screen-printed electrodes. The RSV fusion protein was captured in a sandwich by a specific monoclonal antibody attached to the polystyrene surface and a second antibody-coupled with HRP. The enzymatic reaction HRP-TMB was traduced into an amperometric signal by the electrode. The whole test needed only 25 min to produce result showing similar selectivity and sensibility to commercial RT-PCR or immunofluorescent tests when probed on clinical samples.

Perez et al. [139] described the detection of RSV by nanoparticle amplification of immuno-PCR assay. Their test detects RSV by viral particles capturing into a sandwich formed between two different antibodies specific against the F protein. The first antibody was attached to magnetic beads to allow extraction, while the second was co-immobilized onto gold nanoparticles with specific DNA sequences. After extraction the hybridized DNA can be released by heating and detected via RT-PCR. It was shown that this nanoparticle amplified immune-PCR assay not only provided better virus sampling but also reduce the effect of PCR-induced variation in sample replicates by increasing the tag DNA concentration and by lowering the background signaling. Compared to ELISA and RT-PCR detection, the nanoparticle amplified immune-PCR showed a several 1000-fold improvement in the limit of detection as it detected 4.1 PFU/mL.

## Conclusions

**Figure 10:**
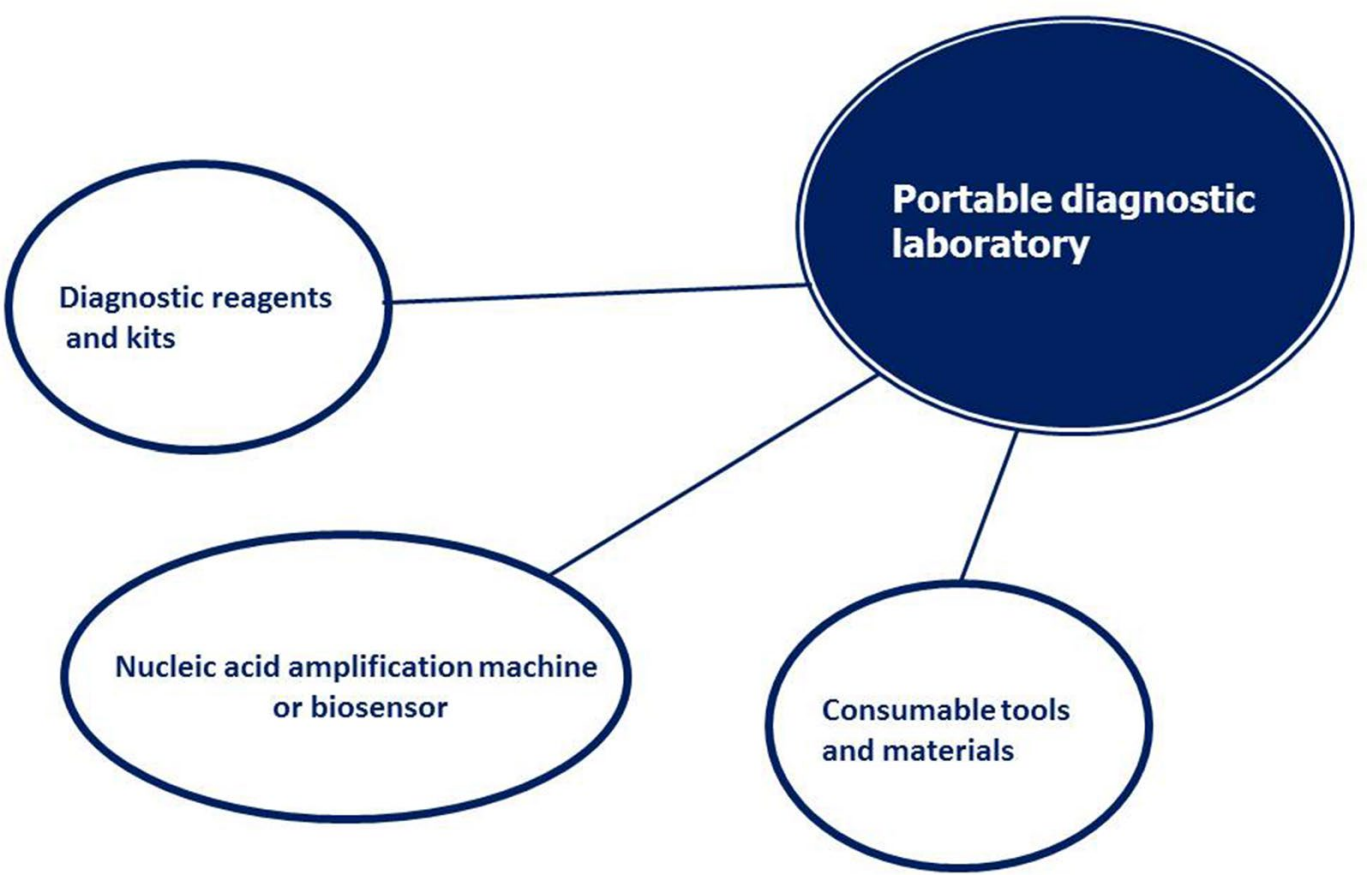
**Elements of integrated portable diagnostic laboratory.** Advanced analytical technologies will offer to veterinary practitioners a suitcase containing consumables, reagents and devices to perform in field diagnostics with laboratory-grade performance.

We show some advanced biotechnological approaches that allow early detection of pathogens that affect domestic livestock and poultry. The pathogens presented in this review entail significant economic losses due to weaken food production systems, increased veterinary costs and may pose a direct threat to global food security. In addition, infection diseases of animal population carry public health risks of outbreaks as well as sporadic and endemic zoonoses. Conventional diagnostic techniques are frequently time consuming, labor intensive and require to be performed on sophisticated equipment by trained professionals with certain experience. New technologies are employed to overcome these difficulties and to provide accurate, simple and affordable biosensor devices that can give rapid response to test. Biosensors have been designed to detect the target (protein or nucleic acid sequence) related to the pathogen by using sensitive and selective recognition properties of antibodies, aptamers, glucans and DNA probes. These sensing elements are associated with a transducing element (electrochemical, optical or colorimetric) that emits a direct signal when the target is recognized. Although most of presented analytical devices are used only in research laboratories, it is expected that more biosensors will emerge in the future given the rapid spread of livestock infectious diseases. There is a strong need to develop portable, miniaturized and multitargeting devices that can be used directly in the field by veterinaries or by competent national authorities responsible for organizing disease controls. To be recommended by the World Animal Health Organization, biosensors for animal infectious disease diagnosis have to undergo inter-laboratories testing to be harmonized and validated. To be used outside research laboratories, a biosensor usually needs adaptation for implementation in field conditions in order to reduce the risk of obtaining false positive or negative results. Among presented detection strategies, the nucleic acid-based biosensors appear being highly suitable for swift and sensitive testing. Their limitation lays in sample preparation steps that include nucleic acids extraction and amplification. The analytical properties of antibody- and aptamer-based biosensors depend strongly on selectivity of these biomolecules and also on the stability obtained after their immobilization on the sensor surface. Furthermore, over the long term, we believe that biosensors technology combining nanotechnologies, advances nucleic acid amplification methods, and next-generation sequencing analysis will be a powerful systemic tool for pathogens detection and surveillance system to control animal disease outbreaks and prevention. Development of reference materials, harmonization of sampling methods, mobile analysis and data networking will significantly support developing of high sensitive and selective biosensors for real-time in field monitoring (Figure 10). Surveillance big data obtained from those diagnostic technologies will help disease prevention and pathogens control to livestock and poultry industrials and will improve animal welfare.
